# Innovating Respiratory Diagnostics: The Game-Changing Role of Biomarkers

**DOI:** 10.3390/jcm13195850

**Published:** 2024-09-30

**Authors:** Silvano Dragonieri, Andras Bikov

**Affiliations:** 1 Respiratory Diseases, University of Bari, 70121 Bari, Italy; 2 Manchester University NHS Foundation Trust, Wythenshawe Hospital, Manchester M23 9LT, UK; andrasbikov@gmail.com

The field of respiratory disease diagnostics has been propelled forward by recent innovations in biomarker research. As the prevalence of chronic respiratory conditions such as obstructive sleep apnea, COPD, asthma, bronchiectasis, and interstitial lung disease continues to rise globally, there is an increasing demand for non-invasive, accurate, and early diagnostic tools [1,2]. This Special Issue on Biomarkers and Diagnostics in Respiratory Diseases brings together pioneering research that highlights the growing potential of novel biomarkers. Through the application of these biomarkers, clinicians can diagnose respiratory diseases earlier and more accurately, paving the way for better-targeted treatments and improved patient outcomes.

In this regard, Gwadera et al. [3] provided insights into calcium and phosphate metabolism in sarcoidosis, aiming to connect these elements with disease activity. While calcium levels were higher in sarcoidosis patients than in healthy controls, phosphate was more significantly correlated with quality of life. These findings indicate that alterations in calcium and phosphate levels could be useful biomarkers to assess disease activity and should be routinely assessed in clinical practice [3].

Hansen et al. [4] introduced the biomarker CPa9-HNE, a serological neoepitope reflecting neutrophil elastase activity, which showed superior diagnostic capability in differentiating between COPD, idiopathic pulmonary fibrosis, and healthy participants compared to traditional calprotectin measurements. The study demonstrated that CPa9-HNE could serve as a valuable tool for early diagnosis, particularly given its association with neutrophil-driven inflammation, which is pivotal in both COPD and IPF [4].

The small airways are traditionally regarded as essential sites of airway inflammation in both severe asthma and COPD. Carpagnano et al. [5] examined patients with severe asthma and small airway disease (SAD) using impulse oscillometry (IOS) to evaluate airway resistance. Their results emphasized the efficacy of single-inhaler triple therapy, which improved lung function and reduced inflammation, measured by the decrease in exhaled nitric oxide (FeNO) levels. These findings suggest that controlling SAD may help achieve stable asthma management and potentially avoid the use of biological therapies [5].

Inhaled corticosteroids are generally discouraged in patients with bronchiectasis, unless they exhibit an eosinophilic endotype. However, it is often difficult to assess airway eosinophilia. Campisi et al. [6] explored the role of FeNO in identifying inflammatory patterns in patients with bronchiectasis and its overlap with asthma. The study revealed that FeNO could serve as a non-invasive marker to detect eosinophilic inflammation, offering clinicians a tool to better understand and manage bronchiectasis appropriately [6].

The L-arginine/dimethylarginine/nitric oxide pathway is a widely investigated topic that can link lung diseases with cardiovascular disorders. Hannemann et al. [7] studied asymmetric (ADMA) and symmetric dimethylarginine (SDMA) in people with chronic airflow obstruction (CAO) and obstructive sleep apnea (OSA). Their study revealed significant perturbations in the ADMA and SDMA levels in individuals with CAO, with or without OSA. These findings indicate that ADMA and SDMA may serve as promising biomarkers to assess cardiovascular consequences in patients with CAO and provide additional insight into the overlap between OSA and CAO [7].

The collective findings in this Special Issue underscore the transformative potential of biomarkers in respiratory disease diagnostics. Biomarkers such as CPa9-HNE and ADMA/SDMA offer new possibilities for early diagnosis, personalized treatment, and disease monitoring. Non-invasive biomarker-based tests, such as those using exhaled breath and sputum [1,2], will likely become increasingly important in the management of chronic respiratory diseases, reducing the need for invasive procedures such as biopsies and providing real-time insights into disease progression.

In conclusion, the research presented in this Special Issue highlights the critical role that biomarkers will play in the future of respiratory disease diagnostics. From asthma and bronchiectasis to COPD and sarcoidosis, novel biomarkers hold the potential to significantly improve disease management and patient outcomes. As we continue to refine these tools, clinicians will be better equipped to deliver personalized and effective care to patients suffering from respiratory diseases.

## Data Availability

No new data were created for this editorial.
